# Recommendations for a Dutch Sustainable Biobanking Environment

**DOI:** 10.1089/bio.2021.0011

**Published:** 2021-06-09

**Authors:** Rogier van der Stijl, Peggy Manders, Elisabeth W.H.M. Eijdems

**Affiliations:** ^1^ University of Groningen, University Medical Center Groningen, Groningen, The Netherlands.; ^2^ UMCG Research BV, University Medical Center Groningen, Groningen, The Netherlands.; ^3^ BBMRI.nl, Biobanking and Biomolecular Resources Research Infrastructure, The Netherlands.; ^4^ Radboud Biobank, Radboud University Medical Center, Nijmegen, The Netherlands.

**Keywords:** biobank, sustainability, policy recommendations, macro-environment, overarching challenges

## Abstract

Biobanks and their collections are considered essential for contemporary biomedical research and a critical resource toward personalized medicine. However, they need to operate in a sustainable manner to prevent research waste and maximize impact. Sustainability is the capacity of a biobank to remain operative, effective, and competitive over its expected lifetime. This remains a challenge given a biobank's position at the interplay of ethical, societal, scientific, and commercial values and the difficulties in finding continuous funding. In the end, biobanks are responsible for their own sustainability. Still, biobanks also depend on their surrounding environment, which contains overarching legislative, policy, financial, and other factors that can either impede or promote sustainability. The Biobanking and Biomolecular Research Infrastructure for The Netherlands (BBMRI.nl) has worked on improving the national environment for sustainable biobanking. In this article, we present the final outcomes of this BBMRI.nl project. First, we summarize the current overarching challenges of the Dutch biobanking landscape. These challenges were gathered during workshops and focus groups with Dutch biobanks and their users, for which the full results are described in separate reports. The main overarching challenges relate to sample and data quality, funding, use and reuse, findability and accessibility, and the general image of biobanks. Second, we propose a package of recommendations—across nine themes—toward creating overarching conditions that stimulate and enable sustainable biobanking. These recommendations serve as a guideline for the Dutch biobanking community and their stakeholders to jointly work toward practical implementation and a better biobanking environment. There are undoubtedly parallels between the Dutch situation and the challenges found in other countries. We hope that sharing our project's approach, outcomes, and recommendations will support other countries in their efforts toward sustainable biobanking.

## Introduction

Biobanks enable the collection, management, storage, and use of human biomaterials and associated data for research purposes. Many different biobank forms^1,2,3,4^ and definitions^5–7^ exist, and as a result, the term biobank can be interpreted differently by different stakeholders.^8,9^ In this article, we follow the new ISO 20387:2018 biobanking standard^10^ and the Minimum Information About BIobank Data Sharing (MIABIS) definition^11^ where, in short, a biobank is the organization or infrastructure that performs the activity of biobanking. A biobank can subsequently contain one or more defined collections of biomaterials and associated data. The biomaterials, data depth, and statistical power these biobank collections provide are considered essential for answering many contemporary research questions.^12^ In fact, in cancer research, between 40% and 50% of publications are estimated to include human biomaterials or their derivatives.^13,14^ Overall, biobanks are lauded as a critical resource for translational research and a way toward more personalized medicine.^4,15,16^

Collecting biomaterials and associated data and the subsequent scientific research is a time-consuming process. Although scientific publications often start to accumulate after the first few years, there remains a considerable lag between the creation of a biobank collection and its expected return on investment through new knowledge, innovations, and patient impact. During this time, the biobank or collection needs to operate in a sustainable manner, to prevent a waste of samples, data and, often, public funds. Sustainability can be defined as *the capacity of a biobank to remain operative, effective, and competitive over its expected lifetime*.^17^ Sustainable biobanking encompasses many different aspects, which can be classified in a framework of three partly overlapping dimensions: (1) the operational dimension (e.g., internal biobank processes, quality management), (2) the social dimension (e.g., relationship with stakeholders, community standards), and (3) the financial dimension (e.g., resources, costs).^18^ Failure in one of these dimensions will lead to a biobank that is no longer operative, effective, and/or competitive; hence, not sustainable.

Sustainable biobanking remains a challenge; in part because biobanks operate in a complex and dynamic environment.^18^ Biobanks act at the interplay of ethical, scientific, and commercial values, balancing both societal and research expectations. In this multidisciplinary environment, a biobank's sustainability is constantly challenged by technical, logistical, legal, and privacy-related issues and a growing demand for quality, FAIRification,^19^ transparency, and accountability.^18,20,21^ Ensuring the long-term involvement of biobank participants also requires continuous effort,^22^ and for many biobanks, a major hurdle is acquiring sufficient funding.^23–26^ Biobanks and collections are foremost responsible for their own sustainability. However, they also depend on their surrounding environment, which contains overarching legislative, policy, financial, and other factors that can either impede or promote sustainability.

Within Work Package 6 *Sustainable and Interactive Biobanking* of the Biobanking and Biomolecular Research Infrastructure of the Netherlands (BBMRI.nl)—the Dutch node of the BBMRI European Research Infrastructure Consortium (BBMRI-ERIC)—we worked on improving the sustainability of individual biobanks and on creating the right environment for sustainable biobanking. To support individual biobanks, we drafted guidelines^27^ and gathered good practices,^28^ business tools,^29^ and background knowledge^30^; all based on recent literature, available case studies, and biobank workshops.^25^ These are publicly available on www.bbmri.nl/sustainable-biobanking and the Biobank Learning Platform of the International Agency for Research on Cancer (https://learning.iarc.fr/biobanking/) and were presented at the Europe Biobank Week 2020.

In this article, we present the final outcomes of our project to improve the environment for sustainable biobanking in the Netherlands. First, we describe the current Dutch overarching challenges for sustainable biobanking. These challenges were gathered during workshops with Dutch biobanks and data infrastructures and during focus groups with biobank users from academia and industry, of which a full description can be found in separate BBMRI.nl reports.^12,25^ Second, we present our recommendations for creating an environment that stimulates and enables sustainable biobanking in the Netherlands. We believe our approach and outcomes are useful for the international biobanking community, bearing in mind the unique circumstances in each country.

## Current Dutch Overarching Challenges for Sustainable Biobanking

From all Dutch scientific fields, most public research and development (R&D) spending is in the field of medical and health sciences; accounting for €1.6 billion out of a total of €5.6 billion in 2018. Furthermore, this field has also seen the sharpest increase in public R&D spending compared with other fields of science.^31^ Perhaps as a result of these investments, the Dutch biobanking landscape is extensive for a country of just ca. 17.5 million inhabitants.^32^ The Netherlands harbors at least 400 clinical and population-based biobank collections.^33^ Most biobanking activities take place at the eight Dutch University Medical Centers (UMCs) and the Netherlands Cancer Institute (NKI). In 2009, the Dutch biobanking field spearheaded by the UMCs, the clinical biobank initiative Parelsnoer^34,35^ and the population biobank LifeLines,^36^ joined forces in BBMRI.nl.^37^ It is BBMRI.nl's mission to maximize the use of samples, images, and data for health research through harmonization and FAIRification of Dutch biobanks. In 2016, the European Population Imaging Infrastructure (EPI2) and the Center for Translational Molecular Medicine—Translational Research IT program (CTMM-TraIT) joined as well.^38^ Since 2019, BBMRI.nl has moved toward integration into Health-RI, the overarching Dutch health data research infrastructure.^39^

However, despite continuous efforts of BBMRI.nl and other stakeholders, Dutch biobanks and their users still experience challenges on a number of overarching topics.

### Sample and data quality

Sample and data quality is an essential aspect, underpinning all biobank research outcomes and thus its main value. In the Netherlands, most UMCs have a centralized biobanking infrastructure, which takes care of sample management, storage, and distribution according to certain quality standards. Collection and processing of the samples and data can be arranged differently for each individual biobank or collection. For the 18 federated Parelsnoer collections,^35^ collection and processing procedures for most sample types have been harmonized. These can be considered as the first steps toward ensuring comparable quality between the Dutch UMCs.

Despite all these improvements by biobanks, in practice it can still be difficult for individual researchers to determine the actual quality of collected biomaterials and data.^12^ Incomplete or missing meta-data on the preanalytical processing and/or usage history seems to be a common issue.^12,25^ Especially meta-data on the preanalytical phase is necessary to evaluate if a sample is fit for purpose. Variations in this phase can change the sample analytes, leading to irreproducible results.^41^ Also, sample quality can be quite variable between biobanks and collections hosted at different organizations, due to different standards and/or procedures for their collection and processing. Both the missing meta-data and the divergent standards can negatively impact the reproducibility of research results, influencing the trust stakeholders have in biobanks and biomedical research.^25,42^

Next to variation between biobanks, there is also a discrepancy between academic, clinical, and industrial environments on standards and requirements. It is not uncommon that, for example, a biomarker found in a high-quality academic collection cannot be reproduced during clinical validation in samples obtained through routine care processes, which are often of a more variable “real world” quality due to less strict procedures.^25^ In addition, the translation of research results into new therapies—and thus patient impact—is complicated by the differences between industry and academia. Industry has to comply with strict regulatory standards which prescribe a high level of documentation, including preanalytical meta-data.^43,44^ Academia generally follows less strict standards, resulting in insufficient levels of documentation.^12^ As a result, many samples and data collected in academic biobanks cannot be used in commercial R&D projects, even though the intrinsic quality of the samples could be fit for purpose. Such differences lower the potential translational impact of biobanks.

### Funding

Funding is seen as a major challenge.^23–26^ The topic concerns both the funding for biobank infrastructures and for the maintenance, use, and reuse of existing collections. Overall, the current funding landscape is not favorable for biobanks, as most research funding is awarded on a competitive, often relatively short-term, project basis, with a focus on new research.^1,25^ In addition, as the number of biobanks and collections grows, competition on this project-based research budget will only increase. Consequently, biobanks and collections experience a short funding horizon (i.e., time until new funding is needed). As a result, their focus shifts more toward short-term *ad hoc* actions instead of measures that support long-term sustainability. In addition, the short funding horizon can prevent stakeholders from giving their commitment and collaboration, as they are uncertain about the future survival of a biobank or collection.^25^

There are, however, also positive developments. ZonMW, a Dutch funding agency for health research, has already made the reuse of existing research infrastructures mandatory for particular grant applications.^45^ In addition, the Dutch Cancer Society runs specific calls for research infrastructures.^46^ Also, most Dutch research institutes acknowledge the essential role of biobanking and structurally fund a centralized biobanking infrastructure. The current movement within the national and international funding landscape toward impact, reuse, FAIR, and open science should contribute to new opportunities.

### Use and reuse

There are concerns about the underutilization of biobanks.^2,23,47^ Many biobanks in a recent survey reported utilization rates of less than 10%.^48^ Low utilization rates can limit the potential impact and overall value of a biobank; both topics which are relevant for sustainability.^49^ How to reconcile such findings on underutilization with a US National Cancer Institute study claiming that 63% of Population Science Cancer Research grants were using already existing biomaterials?^14^ In the Netherlands positive examples of biobank utilization exist, such as the Nijmegen Biomedical Study^50^ and the Dutch National Tissue Portal, a collaboration between BBMRI.nl and The PALGA Foundation to unlock the tissue archives within Dutch pathology laboratories.^51^

There can be multiple causes for insufficient use, some of which are on the level of the individual biobank or collection (e.g., irrelevant samples/data, limited marketing) or outside anyone's influence (e.g., scientific advancements, changing technologies). One reason is that researchers, an academic biobank's main user group, are generally quite low on resources (i.e., cash), limiting their ability to pay possible sample and data issuance costs. In addition, low use is, in part, inherent to the current scientific system, which promotes new work and not the validation of previous research or the reuse of existing samples, data, standards, and infrastructures.^25^ Furthermore, by promoting competition between researchers on research funding and rewarding publications, the current scientific system contains limited direct incentives for researchers to share collected samples and/or data with others. This can result in protectionism, as researchers want to get publications and research funding out of their collections. Another reason for a lack of sharing is that researchers often spend years building a collection or biobank. The result can be an understandable sense of entitlement where researchers see the collected samples and data as *their* samples and data, even when the collection is publicly funded and participants have given their informed consent for broad use. Lastly, the Not-Invented-Here Syndrome^52,53^ is also likely to contribute. The associated bias against external parties limits the reuse of samples and data from nonlocal biobank collections.

At many biobanks, there is room to improve accessibility and transparency.^12,25,54^ Clear access procedures, uniform policies on ethical reviewing and clarity on privacy, data protection, and international data sharing rules would greatly shorten procedures and prevent frustration and cancelled research projects. The variability between Dutch research institutes in their access policies, contracting, and IP approaches complicates the use of multi-institutional biobanks, especially in public–private partnerships. Also, applicants often need separate approval from each local Ethics Review Committee.^12^ This results in long issuance procedures with plenty of opportunity for delay.

Findability is an important aspect of use. Both academic and industrial users expressed difficulties in finding the samples and data they need.^12^ In an effort to increase findability, BBMRI.nl continuously invests in the national sample and data catalog^33^ and the request portal PODIUM.^40^ Improving findability by keeping the catalog up to date and increasing awareness of its existence is an on-going effort.

### Linkages

Linkages between biobanks, national registries, and other data sources allow new research questions to be answered, enhancing the potential value of collected samples and data. This also increases their potential for reuse. Samples linked to clinical and phenotypic data seem to be in demand, especially for industrial parties.^12^ There is sufficient room for improving the efficiency and quality of linkages. Creating linkages is often technically doable. However, legal and privacy-related issues and the lack of a unique personal identifier for research purposes make linking different data sources a tedious process.^55^ Even though the Netherlands is not yet at the level of countries such as Sweden or Estonia, progress has been made in recent years, linking Dutch biobanks to large data registries such as the Dutch Cancer Registry, PALGA, and Statistics Netherlands.^56^ Overall, the Dutch health and socioeconomic registry landscape is broad and of high quality, containing around 150–200 health and/or patient registries for potential linkages.^57,58,59^

### Image

Biobanks depend on the support and trust of many different stakeholders; from participants to funders.^18,60^ As such, the overall image that these stakeholders have of biobanks greatly affects sustainability. According to researchers, biobanks are essential for answering many modern research questions.^12^ In addition, the fact that participants still donate their samples and data, and funders still fund new initiatives is at least indicative of their positive view of biobanks as added value for science and society. However, the high costs,^61–63^ reproducibility issues,^41,42^ and protracted return on investment do not contribute to a positive image. This can prevent key stakeholders from giving their long-term commitment. Also, an often-heard comment is that biobanks and associated researchers are more interested in collecting samples than in actually using them; which seems to be supported by the reported low utilization rates.^48^ In the end, a poor overall image of biobanks and biobanking can be both a cause and a consequence of limited sustainability, creating a vicious cycle that can be hard to break.

## Recommendations for Creating the Right Conditions for Sustainable Biobanking

**FIG. 1.**
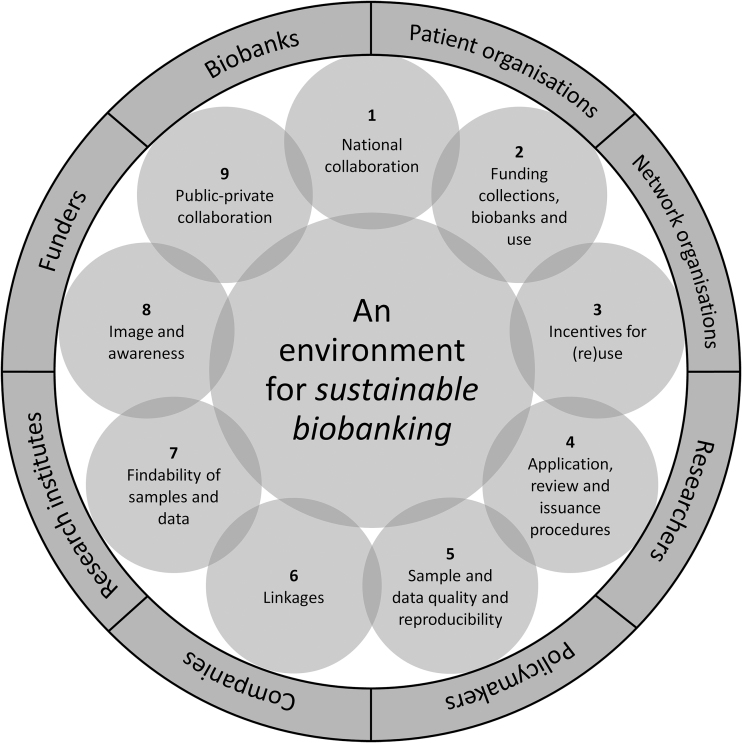
Recommendations for a sustainable biobanking environment across nine themes. To create an environment for sustainable biobanking in the Netherlands steps have to be taken on nine themes. Each theme contains one or more recommendations (Table 1). Eight stakeholder groups play a central role in translating these recommendations into practice.

To address the abovementioned overarching challenges and create an environment that enables sustainable biobanking, we drafted recommendations across nine themes (Fig. 1 and Table 1). The recommendations are based on input gathered during biobank workshops^25^ and focus groups with biobank users,^12^ supplemented with relevant literature, specific expert input (see Acknowledgments section), and the authors' experiences. With these themes and recommendations, we give directions. Translating them into daily practice will be a multistakeholder effort. What specific stakeholders to involve and what their role should be differs per recommendation and per country. As such, we have limited our discussion of specific stakeholders in the text, but do provide suggestions of stakeholder groups to involve per the recommendation in Table 1.

**Table 1.** Recommendations for Improving the Preconditions for Sustainable Biobanking in the Netherlands

Theme	Recommendations	Biobanks	Companies	Funders	Network org.	Patient org.	Policymakers	Researchers	Research inst.
1. National collaboration	Form a national collaborative platform for the biobanking community focused on operational topics, tackling overarching challenges and connecting local communities with national and international developments	•			•				•
2. Funding collections, biobanks, and use	Create awareness that academic collections and biobanks are not self-sustaining	•			•			•	
	Provide structural funding for central biobank infrastructures within the larger research institutes	•		•					•
	A more prominent role for funders in promoting sustainable behavior in new and existing biobanks and collections			•					
	Draft a business plan before starting a collection or biobank	•		•				•	
	Create a funding stream toward biobanks based on use, hereby creating an incentive to share			•					
	Fund FAIR and Open Science			•					
3. Incentives for use and reuse	Create incentives that reward sharing and (re)use and make shielding collections unfavorable			•			•		•
	Set funding conditions that stimulate the (re)use of collections			•					
	Ensure clear recognition of a biobank and/or collection in scientific publications to link value to (re)use	•			•				•
	Involve nonscientific stakeholders in collections and biobanks to emphasize societal interests	•		•	•	•		•	
	Unburden researchers to lower their sense of entitlement	•							•
	Educate current and future researchers on the societal and individual benefits of collaboration, sharing, and reuse	•			•	•			•
4. Application, review and issuance procedures	Make sure application, assessment, and issuance procedures are transparent and accessible	•						•	•
	Create national policy concerning biobank research, specifically for The Netherlands: Create national policy concerning research not covered by the Dutch Medical Research Involving Human Subjects Act.				•		•		•
	Work on shared medical ethical review frameworks between research institutes				•				•
	Improve harmonization of procedures between different research institutes				•				•
	Create national policy on incidental findings	•			•	•			•
	Draft national informed consent guidelines	•	•		•	•			
	Clear policy for European and international sharing of samples and data				•		•		•
	Implementation of the national standard Material and Data Transfer Agreements	•			•				•
	Active support for users on regulations, policies, and procedures when requesting samples and data	•							•
	Make collections use a central biobank, which ensures uniform and simple procedures and user support								•
5. Sample and data quality and reproducibility	Make sample and data quality and reproducibility one of the themes within the national collaborative platform	•							
	Ensure a certified central biobank infrastructure within each research institute that safeguard quality standards	•							•
	Streamline sample and data preanalytics as much as possible with existing standard diagnostic and care processes	•						•	•
	Each collection takes care of correct and complete meta-data, according to national minimal meta-dataset standards							•	
	Connect (financial) incentives to correct and complete meta-data registration	•		•					•
	Ensure that data and meta-data can be registered directly at the source	•	•		•		•	•	•
	Make sure that biobank data standards align with standards used in health care, specifically for The Netherlands: join the National Health Information Council, which sets national standards for information in health care	•			•				
	Raise awareness among researchers about sample and data quality, including the potential risks and consequences	•			•				•
	Ensure that collections and biobanks use an approved data management plan	•			•				•
6. Linkages	Make national agreements on a unique identifier (“key”) for linking in the context of scientific research				•		•		•
	Make national agreements and/or guidelines about the legal frameworks for linkages				•		•		•
	Implement a common national processor agreement for sharing data with third parties	•			•				•
	Make visible which data sources can be linked, including the associated conditions	•						•	•
7. Findability of samples and data	Improve communication about the available catalogs and emphasize their wider importance to all stakeholders. Involve the central biobank infrastructures as a linking pin to the local communities of researchers and collections through the collaborative platform.	•			•				•
	Create local catalogs within the research institutes linked to the national catalog, according to common standards.	•			•				•
	Make findability part of the funding conditions for collections and biobanks and link catalog updates to the periodic grant reporting.			•					
8. Image and awareness	Increase awareness and transparency regarding biobanks toward the general public to maintain support	•			•	•		•	•
	Show the scientific and societal value of collections and biobanks	•			•	•		•	•
	Train the next generation of users by including research infrastructures, ELSI, (re)use, FAIR and Open Science within relevant biomedical curricula	•			•				•
9. Public–private partnerships	Draft a national model agreement for public–private partnerships between biobanks and companies, similar to the Dutch clinical trial agreement	•	•		•				•
	Create an Industrial Liaison Office that stimulates public–private (re)use and collaboration		•		•				•
	Draw up a list of conditions that samples and data must meet if they are to be used for public–private partnerships	•	•		•				
	Develop different models for public–private collaborations between companies and biobanks	•	•		•				
	If aiming for public–private biobank collaborations, make sure private parties are involved at an early stage	•	•					•	

### Theme 1: National collaboration

We recommend the formation of a national platform for the biobanking community, with an infrastructural approach and management to offset personal and institutional interests. The platform should focus on strategic and operational topics, solving overarching challenges, connecting local communities with national and international developments, and creating synergies between institutes and biobanks. The centralized biobanking infrastructures within the larger research institutes are a good foundation for such a collaborative platform. Most overarching challenges can only be solved through multistakeholder collaboration. A national biobanking platform can be the connecting factor and play a major role in involving other stakeholders and driving the other themes and associated recommendations forward.

### Theme 2: Funding collections, biobanks, and use

Funding is one of the larger challenges for biobanks. To support biobanks in this and other challenges, we drafted a separate set of guidelines for sustainable biobanking aimed at individual biobanks.^27^ One thing biobanks should do is draft a business plan, together with key stakeholders, before starting the biobank, as this seems to support sustainability.^28,64,65^ In addition, biobanks should create awareness among their stakeholders that academic biobanks and their collections will, in general, not be self-sustaining. A maximum cost recovery of 5%–25% appears realistic.^66–68^ As such, additional external funding sources will always be required.

On an overarching level, we first recommend dedicated core funding for the centralized biobanking infrastructures within the larger research institutes. These biobanking infrastructures form the backbone of the Dutch biobank landscape by bundling expertise, connecting local researcher communities, enabling quality, and creating economies of scale. Structural funding of these research infrastructures offers continuity to all stakeholders involved and indirectly supports the sustainability of the individual collections they host. A minimum level of structural core funding should be a shared effort between the individual hosting institutes and national infrastructure program funders.

Second, we recommend a more prominent role for research funders in promoting sustainable behavior in new and existing biobanks and collections. For example, funders could make a business plan and a market analysis mandatory in funding applications; make reuse part of the funding requirements and progress reports; and reward the reuse of existing collections in the assessment of research proposals. Furthermore, we recommend setting up a use-based funding stream toward biobanks, creating a direct incentive for sharing and reuse. An option would be to issue specific “use” calls, such as the EIT Health Digital Sandbox call.^69^ Reuse does not only benefit biobanks but also the research funders, and through them society itself. Research by the US National Cancer Institute shows that population-based research projects using existing biomaterials were 4.2 times less expensive, leaving more money for research itself. In addition, their output was higher with 1.4 times more publications per year than projects that collected new biomaterials.^14^

### Theme 3: Incentives for (re)use

We need to create incentives that reward sharing and (re)use and make the shielding of collections disadvantageous. This will be difficult, requiring the collaborative effort of hosting institutes, funders, and policymakers, among others. An option could be to make the contributions to, use of and impact created by biobanks, collections and similar research infrastructures part of the assessment of researchers and research institutes. In addition, we need to agree on a system for the unambiguous recognition of biobanks in scientific publications so that sharing contributes to scientists' careers. Internationally, efforts have been made^70,71^; Dutch institutions, network organization, and biobanks need to implement and enforce them. Active sharing could also be part of the funding conditions and reporting, as mentioned in Theme 2: Funding Collections, Biobanks, and Use section. Finally, we also recommend actively involving societal stakeholders in the governance of biobanks, to provide a counterweight to personal, institutional, and scientific interests.

### Theme 4: Application, review, and issuance procedures

We recommend improving the application, reviewing, and issuance procedures both on the level of the individual biobanks and on the national level. Nationally, institutes, network organizations (e.g., BBMRI.nl, Health-RI), and policymakers need to work on shared frameworks for medical ethical reviewing across biobanks and research institutes. The decision of a local Ethics Review Committee should be accepted by Review Committees in other institutes. This will greatly improve the accessibility of multicenter collections. Matching our recommendations, the Dutch Minister of Health, Welfare, and Sport commissioned an evaluation of the Dutch ethics reviewing system in medical research, including biobank research, involving a broad representation of stakeholders.^72^ In addition to harmonizing national reviewing policies, centralization of the application, reviewing, and issuance procedures within each UMC would simplify and accelerate processes. Using digital request portals such as PODIUM^40^ or the BBMRI-ERIC Negotiator^73^ would further support reuse. Furthermore, central biobanking infrastructures need to collaborate more closely to align procedures, preferably through the national biobanking platform as mentioned in Theme 1: National Collaboration section. On an international level, a clear policy for the sharing of samples and data across national borders is called for.

### Theme 5: Sample and data quality and reproducibility

Samples and data of comparable quality and with correct metadata (e.g., preanalytics, storage, issuance, analysis, patient background, and treatment) are essential to match collections and verify findings. We recommend, as part of the national biobanking platform (Theme 1: National Collaboration section), to set up national working groups on quality and reproducibility. These working groups should strive for the implementation of harmonized evidence-based protocols for sample and data collection across institutes; improve awareness about the role of the preanalytical phase among researchers, and draft a national minimal metadata standard for biobanks and collections. Collaboration with relevant national and international platforms and initiatives is crucial. Furthermore, on the topic of metadata, research funders could make complete and correct metadata registration part of their funding conditions or create a direct financial incentive. To reduce variability between collections and reduce reproducibility issues during clinical translation, we also recommend biobanking to be integrated in standard diagnostic and clinical procedures as much as possible.

### Theme 6: Linkages

Linking different data sources makes it possible to answer new scientific questions. Currently, making linkages is a lengthy process, in which the nontechnical subjects (e.g., legal, agreements, standards) are the greatest obstacles. We recommend agreeing on a national unique identifier to facilitate linkage of datasets in the context of scientific research. In the Netherlands, discussions have been ongoing for several years on using the Dutch Citizen Service Number for research-related linkages.^74^ So far, the Dutch Government has not been willing to make this legally possible, despite continuous lobbying from the field. In addition, we recommend making national agreements and/or guidelines on the legal frameworks concerning linkages between biobanks and other data sources. In addition, implementation of a national common processor agreement for data sharing with third parties will prevent individual legal interpretations between organizations, resulting in faster turnaround times.

### Theme 7: Findability of samples and data

To promote use and prevent duplication of efforts, available samples and data must first be findable. In general, findability is supported by national and international meta-data catalogs and design papers. Usually, the responsibility for including and updating the information in such catalogs lies with individual collections. To improve the information in these catalogs, research institutes should create local master catalogs, according to national agreements, and link these to existing national and international counterparts. A clear starting point for agreements would be the international MIABIS standards.^11^ The local catalogs should be under management of each institute's centralized biobanking infrastructures. Acting as linking pin toward local communities of researchers and collections should help improve efforts into complete profiles. These local catalogs should be filled automatically from existing sources as much as possible. In addition, we recommend making updating catalogs part of the conditions and progress reports of research funders, related to Open Science and FAIR data. Recent research also suggests that findability through catalogs is not sufficient to increase usage if only meta-data are provided. Researchers look for collaborators and also require information about research excellence (e.g., investigator profiles, cohort output).^75^

### Theme 8: Image and awareness

For their sustainability, biobanks depend on the support of many stakeholders. The same is true for translating these recommendations into practice. A positive image of biobanks is essential and therefore we recommend improving the interaction with and the awareness of the general public and other stakeholders about the scientific and societal value of biobanks and related research. This is in line with the increased focus on public involvement^76^ and return of results.^77^ Furthermore, to better determine biobank value, both biobanks and involved stakeholders should shift their focus from internal biobank measures toward biobank output.^78^ A focus on output with appropriate output measures also permits economic analyses, providing stronger tools for funders and policymakers.^78^ In addition, we recommend educating the next generation of users by addressing biobanks, research infrastructures, FAIR data, and related topics in biomedical curricula, hereby, hopefully also improving future biobank use.

### Theme 9: Public–private collaboration

The potential for public–private collaboration in the field of biobanking is underused, even though there is a demand from companies for samples, data, and research ideas.^12,79^ This lack of cooperation is also undesirable from a societal point of view, as the private sector is generally needed to bring new innovations to the market and create impact for patients. Academic researchers and biobanks may benefit from public–private partnerships through joint publications, enhanced reputation, and visibility, additional funding and the exchange of knowledge.^80^ A common obstacle is that academically gathered samples and data cannot be assessed for commercial R&D use due to limited documentation (e.g., meta-data).^12^ Moreover, the new European Medical Devices Regulation^43^ and *In vitro* Diagnostics Regulation^44^ obligate industry to use well-documented samples for performance testing and validation of diagnostics.^81^ Therefore, we recommend developing guidelines and criteria that academic biobanks should take into account if they plan to collaborate with companies in the future. On the Dutch national level, this could be done by the intended biobanking platform (Theme 1: National Collaboration section) in collaboration with the Association Innovative Medicines, the Dutch representative body of the pharmaceutical industry. On an international level, there could be a role for the BBMRI-ERIC Industry Stakeholder Forum. Furthermore, biobanks should involve industry in an early phase, before actual collection starts, to ensure compatibility, align needs, and share knowledge. Also, a major step forward would be a nationally agreed standard contract for public–private collaborations, similar to the Dutch standard clinical trial contract^82^ that functions as a starting point for negotiations.

## Discussion

In an ideal setting: *biobanks effectively and efficiently facilitate biomedical research, leading to scientific breakthroughs and societal impact. Biobanks provide a supply of high-quality and well-annotated samples linked to extensive datasets, while safeguarding the participant's interests. Participants have control over the use and reuse of their samples and data and are actively informed about the impact of research. The power and data depth of linked biobank collections allow new research questions to be answered. Collections are accessible across sectors and borders and their reuse is incentivized. Furthermore, biobanks are an integrated part of biomedical research institutions, bridging research and the clinic. As a result, biobanks are acknowledged as vital research infrastructures for the transfer of biomaterial from donor to researcher and serve as trusted partners in the midst of biomedical research communities.*

Currently, biobanks are not there yet as sufficient challenges remain, both on an overarching level and on the level of the individual biobank or collection. Their seemingly insufficient use^48^ and on-going sustainability issues raise valid questions about their effectiveness and added value.^83^ In this article, we have provided the final outcomes of our BBMRI.nl project, giving an overview of current Dutch overarching challenges and presenting recommendations to create an environment that contains policy, legal, financial, and organizational factors to enable and promote sustainable biobanking. Many of the identified challenges and suggested solutions will sound familiar.^84–88^ As such, other countries will undoubtedly recognize many of the challenges listed, of course adapted to their own national circumstances. By sharing our approach and results we hope to support similar efforts in other countries.

The different recommendations and themes are intertwined, affecting one another directly and indirectly. To have impact, progress has to be made on all nine themes. Translating the recommendations toward practical implementation—moving from *what* to *how*—will require the involvement of all relevant stakeholders, including funders, patients, and policymakers. To aid in this process we have indicated recommended which stakeholder groups should, most likely, be involved (Table 1). This translational process will probably identify new challenges, perspectives, and solutions; adjusting what is presented in this study. Therefore, these recommendations should be seen as a starting point or roadmap to bring different stakeholders together in a targeted manner and provide them direction.

Given the need for multistakeholder collaboration, translating these recommendations into daily practice will not be straightforward. It will take time, commitment, and resources. One of the more impactful recommendations is the formation of a national collaborative platform for the biobanking community (Theme 1: National Collaboration section). Such a platform can drive progress on the other themes by bringing together the required stakeholders. This recommendation is already becoming a reality by the establishment of a national Biobank Community under the Health-RI initiative.^89^ The new Biobank Community is both a continuation and an expansion of the work and network of BBMRI.nl. The community will focus on further developing the national biobanking infrastructure by connecting Dutch biobanks and collections and working on shared standards and policies. The community will work in close collaboration with individual biobanks, researchers, patients, funders, policymakers, and other stakeholders. Through the overarching structure of Health-RI, the Biobank Community is closely connected to other thematic communities (e.g., ELSI, Imaging, Omics). We hope this new platform will provide the multistakeholder collaboration, resources, drive, and leadership needed to create the right environment for sustainable biobanking in the Netherlands.

The article has a national perspective. However, several themes have a clear international link and should not be solved by individual countries. One of these themes is sample and data quality and related harmonization and standardization. To allow cross-country comparison of samples and data and ensure reproducibility of research results, we need to establish common international standards and evidence-based protocols across the entire sample workflow. Examples are the quality management work of BBMRI-ERIC^90^ and BBMRI.de^91^ and initiatives such as the new ISO 20387:2018 biobanking standard^10^ and SPIDIA4P, which publishes specific ISO technical standards for the preanalytical phase of sample collection.^92^ From a national perspective we should not reinvent the wheel but make sure we connect to these initiatives and translate them to our own workflows. Still, improving harmonization and standardization has been a topic for years^84,85,87,93,94^; and will be for years to come. Bringing the different worlds of research, clinical care and industry closer together, across country borders, will remain a major challenge.

## Summary and Conclusion

The added value of this BBMRI.nl project lies in providing an overview of the current overarching challenges in the Netherlands and a coherent, shared direction for improvement. Instead of zooming in on a detailed piece of the puzzle we have tried to provide the full picture. By actively involving biobanks and their users, we came closer to their real needs and used their combined creativity to find solutions. Our recommendations provide relevant starting points for all stakeholders to jointly contribute to sustainable biobanking. We hope that sharing our project's approach and outcomes will also support other countries in working toward the right conditions for sustainable biobanking.
